# A Bi-lingual chatbot implementation for pandemic response using the transformer-based approach

**DOI:** 10.1371/journal.pdig.0001256

**Published:** 2026-04-01

**Authors:** Mugume Twinamatsiko Atwine, Daudi Jjingo, Mike Nsubuga, Richard Serunjogi, Ibrahim Mbabaali, Ibra Lujumba, Byansi David, Timothy Kintu, Ronald Galiwango, Kebirungi Grace

**Affiliations:** 1 African Centers of Excellence in Bioinformatics and Data-Intensive Sciences, Kampala, Uganda; 2 The Infectious Diseases Institute, Makerere University, Kampala, Uganda; 3 Department of Computer Science, College of Computing and Information Sciences, Makerere University, Kampala, Uganda; 4 Faculty of Health Sciences, University of Bristol, Bristol, United Kingdom; 5 Jean Golding Institute, University of Bristol, Bristol, United Kingdom; 6 Department of Languages, Makerere University, Kampala, Uganda; Liverpool John Moores University - City Campus: Liverpool John Moores University, UNITED KINGDOM OF GREAT BRITAIN AND NORTHERN IRELAND

## Abstract

The COVID-19 pandemic highlighted the critical need for timely and accurate information in effectively managing pandemics. The proliferation of misinformation on public media platforms complicated the management of the pandemic, necessitating a large and constant human resource to meet the demand for trustworthy information. To address this challenge, we developed an intelligent bilingual chatbot that is available 24/7 to provide medically curated up-to-date and approved pandemic management information in English and Luganda. This approach leveraged deep learning to train a chatbot on a growing corpus of curated pandemic-specific information, questions, and answers. Our results demonstrate an implementation of a chatbot that leverages the well-resourced English NLP framework to enable chatting in the Luganda language. They also show that the chatbot is an effective and flexible tool for disseminating accurate information in real-time, while also providing opportunities for continuous improvement through conversation-driven development.

## Author summary

In the context of a pandemic, optimizing communication and information dissemination is essential for both prevention and timely treatment. The lack of proper information or the proliferation of wrong information can be a major handicap to pandemic management. In this paper, we present a communication-based approach to pandemic preparedness through the design and implementation of a bi-lingual chatbot. It was tailored to meet the specific COVID-19-related healthcare needs of the Ugandan population and can operate in the context of a low-resourced African language - Luganda, which is widely spoken in Uganda. It is based upon a carefully curated selection of information sourced from vetted health authorities and reputable health websites such as the Ugandan Ministry of Health, WHO and the CDC. In principle, this chatbot further provides a blueprint for bi-lingual communication for pandemic contexts beyond COVID-19. As the first of its kind in Uganda, it will hopefully set the stage for leveraging language technologies for pandemic management.

## Introduction

In March 2020, the World Health Organization (WHO) declared COVID-19 as a pandemic, marking a significant global health crisis [1]. The disease rapidly spread across nations, warranting strict public health interventions. Its clinical presentation encompasses several symptoms, including but not limited to headaches, nausea, fevers, and other related manifestations [2]. Since these symptoms are not exclusive to COVID-19 and can vary significantly between individuals, it became difficult to distinguish it from other common illnesses. There arose a considerable amount of confusion about how to understand the disease and its etiology [3], and its transmission risks. Furthermore, its novelty and scale resulted in a dearth of reliable information, paving the way for the proliferation of misinformation on public media platforms [4]. This misinformation, in turn, elicited noncompliance with recommended preventive measures, the adoption of dubious or harmful practices to combat the virus [5], vaccine hesitancy, and mistrust. Consequently, there were several avoidable infections and resultant deaths [6]. Medical personnel were overwhelmed by an avalanche of patients and thus had less time to thoroughly explain the disease to the inquiring public [7].

The need for a dynamic, scalable, and reliable intervention that could collect, synthesize, and present accurate information to the public as and when required was apparent [8]. Several use cases highlight the value of chatbots in healthcare and pandemic management. They are, for example, particularly suitable for COVID-19 screening, minimizing face-to-face interactions when providing information, checking symptoms, and scheduling appointments. They present additional useful attributes like scalability, adaptability, consistent quality responses, and cost reduction, making them an appealing solution in resource-limited settings [9]. While they offer significant promise, multilingual implementation in healthcare contexts presents unique accuracy challenges. Traditional approaches use machine translation to convert queries to a processing language, then translate responses back—introducing potential errors at each step. Recent advances in large language models have accelerated the development of multilingual medical AI systems, with solutions like Apollo [10], MMedLM [11], and Medical mT5 [12] demonstrating different approaches to handling medical knowledge across multiple languages, typically achieving 58–68% accuracy on benchmark datasets through massive multilingual pre-training. Studies such as Multi-OphthaLingua [13] have documented significant performance disparities across languages, with LLMs struggling particularly with clinical content in low-resourced languages.

Nevertheless, they have been shown to be effective in crisis communication, hence assisting the achievement of crisis communication management objectives [14]. Yet this potential of such language technology is underexplored and could be pursued on an inclusive development track that integrates diverse languages and cultures [8]. Indeed, some countries have developed customized chatbots to disseminate context-specific, reliable, and trustworthy information [15]. Such efforts are still constrained by the limited deployment of language technology in several languages, particularly low resource languages [16]. As part of addressing this issue, our chatbot is built to provide information in both English and Luganda, specifically tailored for the Ugandan context. This paper presents an approach to pandemic preparedness through the design and implementation of a bi-lingual chatbot. It was tailored to meet the specific COVID-19-related healthcare needs of the Ugandan population and can operate in the context of a low-resourced African language - Luganda, which is widely spoken in Uganda. Unlike LLM-based systems that rely on massive multilingual pre-training, this approach employs a suffix-based architecture that maintains separate language-appropriate vocabulary, while sharing underlying structures. This task-oriented design, focused specifically on pandemic FAQ and triage tasks rather than general medical knowledge, allows for higher accuracy while maintaining minimal computational requirements. It is thus suitable for deployment in resource-constrained contexts. Its scope, combined with explicit bilingual architecture for English and Luganda, preserves cultural and linguistic appropriateness while ensuring medical content consistency.

To ensure its scalability and versatility, the chatbot was built in an incremental manner that allows for the seamless integration of other cutting-edge AI/ML technologies [16]. It is based upon a carefully curated selection of information sourced from vetted health authorities and reputable health websites such as the Ugandan Ministry of Health and the CDC [17]. Before integration into the chatbot, this information is thoroughly reviewed and analyzed by domain experts, including doctors and clinicians, to ensure its relevance to the Ugandan context. Its implementation uses the DIET (Dual Intent and Entity Transformer) classifier, a multi-task deep learning model that combines intent classification and entity extraction in a single neural network, as the primary algorithm for Natural Language Processing (NLP) [18]. An “intent” represents the purpose or goal behind a user’s message. For example, when a user types “Hello,” the intent is *to greet*. When they ask, “What are COVID-19 symptoms?”, the intent is *to obtain symptom information* (Table 1). This intent and entity identification strengthens its efficiency and accuracy in understanding user input and providing relevant responses. Its architecture is based on transformers, which have been shown to outperform other traditional approaches such as rule-based systems and statistical models [19]. Additionally, DIET has the advantage of handling multiple intents and entities in a single sentence, making it well-suited for complex dialogues that may arise in healthcare contexts [20]. By integrating these language technologies, the chatbot can effectively communicate with users, provide accurate and relevant responses, and continually enhance its performance through machine learning and natural language understanding.

**Table 1:** Classification of user intents with examples illustrating common communication themes in health-related dialogues.

Intent	Examples
Conversational intents	Greetings, farewells, expressions of gratitude
Informational intents	Questions about disease definitions, symptoms, transmission.
Procedural intents	Queries about testing procedures, vaccination processes, treatment protocols.
Locational intents	Finding testing centers, vaccination sites, hospitals.
Safety intents	Prevention measures, protective equipment, isolation guidelines.

## Methods

### Data collection

**Fig 1:**
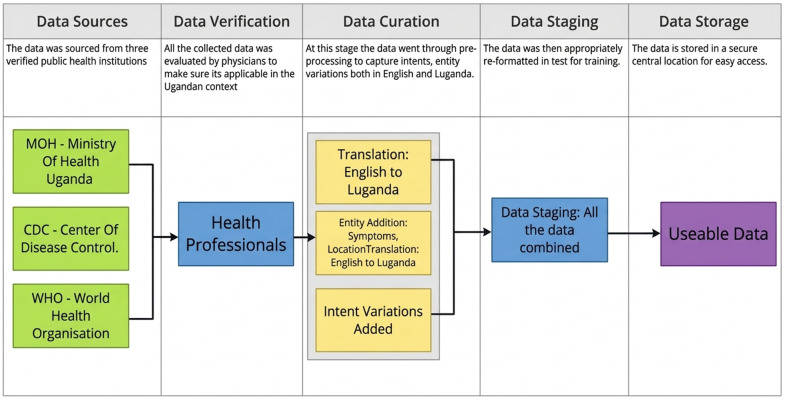
This demonstrates the process through which data was collected, verified, curated, and stored for this project. Data was collected from local data sources such as the Ministry of Health, verified by doctors and curated in English and Luganda before it was stored for use.

The development of this chatbot heavily relied on a foundational dataset to train the NLP algorithm. We collected and curated a comprehensive dataset specifically for training and evaluating chatbots in a pandemic context. We implemented a meticulous data collection approach to maximize the accuracy and dependability of the information from which the chatbot responses would be derived. This involved four major information sources; 1) pandemic guidelines from the Ugandan Ministry of Health [21], 2) the US Centers of Disease Control (CDC) [22], 3) The World Health Organization (WHO) [23] and 4) Ugandan physician-reviewed literature, covering clinical case management, drug dosing, drug reactions, and other pertinent health information. These sources provided largely text data, which was used to formulate responses for Frequently Asked Questions (FAQs) (Fig 1).

### Data curation

Through collaborative consultations, the collected data was carefully reviewed and curated by local health-domain experts and practitioners to ensure not just information validity, but also local relevance and appropriateness. Local relevance criteria included: (1) appropriateness of medical terminology for the Ugandan healthcare system (e.g., referencing locally available medications and treatment protocols), (2) cultural sensitivity regarding health practices and beliefs common in Uganda, (3) alignment with Ugandan Ministry of Health guidelines and protocols, (4) practical applicability given local healthcare infrastructure (testing facilities, vaccination centers, hospital capacity), and (5) addressing specific concerns raised by Village Health Teams during community outreach. For example, information about COVID-19 testing was tailored to reflect the actual testing facilities available in Uganda rather than international protocols that might not be locally feasible. These experts included experienced clinicians, physicians, and members of Village Health Teams (VHTs) who have been actively engaged in IDI’s pandemic responses. By incorporating their invaluable insights, we aimed to fortify our chatbot’s knowledge base, enabling it to provide highly informed and reliable responses to user inquiries. They identified the most common questions, concerns, and misconceptions that people might have about pandemic preparedness and helped proofread the answers. They also added newly available information, for example when the vaccine came online months into the pandemic and the public needed information on types of vaccines, vaccination centers and vaccination outcomes. Together, those various data sources made 505 intents for the initial training of the chatbot (Table 2).

**Table 2:** The 505 initial intents represent unique user goals across both English and Luganda versions. Some intents were implemented in both languages (e.g., greeting and greeting_Luganda), while others were language-specific based on audience needs. (Table 3) shows the final distribution: 375 English FAQ intents and 403 Luganda FAQ intents, with substantial overlap between the two language datasets.

No	Data	Example	Count
1	Questions/Intents	to greet	505
2	Questions Variations	hello, hey, how are you	3,535
3	Entities	Subjects in the data	1,290

The data were also translated into Luganda, a commonly used local Bantu language in Uganda for training the local language capabilities of the chatbot to maximize community outreach. This translated data therefore had similar unique intents as the English data. Due to the multiple possible variations of each intent, we were able to accumulate a dataset of 33,728 (Table 3) data points.

**Table 3:** Intent and data point distribution by language. Data points are variations of each intent (different phrasings, synonyms, misspellings). Different intent counts (374 vs 396) are as a result of a limit we had in resource availability in regard to domain specific translations and variation creation. The 505 initial intents (Table 2) represent combined unique user goals across both languages before language-specific refinement.

Data	No of Intents	Total Data Points	Average Variations
English FAQ	374	20,469	54.58
Luganda FAQ	396	12,235	30.36
English Chit Chat Data	22	204	9.27
Luganda Chit Chat	22	190	8.64
General English	12	43	3.58
General Luganda	18	587	32.61
**Total**	**852**	**33,728**	

### Deriving question variations

The data were then organized into question-answer pairs. For each pair, a unique intent to identify it was added. Whereas the collected intents covered the nature of all substantive questions asked, the chatbot needed to be trained on the various ways in which those intents could be asked. This formed the second level of curation, where each intent was expressed in at least five different ways. The minimum of five variations per intent was chosen pragmatically to balance training data sufficiency with manual curation effort. While larger variation sets improve model robustness, each variation required expert validation for clinical accuracy and local appropriateness. Five minimum variations provided sufficient diversity to capture common linguistic patterns (synonyms, misspellings, different phrasings) while remaining feasible for curation. High-priority intents received additional variations > 5 and the variation set was expanded iteratively based on beta testing feedback. This question variation was based either on the multiplicity of scenarios it could play out and/or simply the linguistic synonyms it could entail. For example, ‘define coronavirus’ could be varied with synonyms for define such as ‘describe coronavirus’ or ‘explain to me coronavirus’. Additionally, different spellings or misspellings for keywords were generated. For example, the different terms casually used to refer to COVID-19 like Covid, covidi, and Sars-cov-2. This generated an initial total of 3,535 question variations arising out of the initial 505 questions (Table 2). This variation step helps maximize coverage by the information database of various ways in which the same entity may be queried by the public. This data was crucial for training and enabling the chatbot to effectively communicate with users in a context-aware way. Again, as for the root questions, these question variations and data were also translated into Luganda, to eventually be used in developing the dual language conversation capacity of the chatbot.

### Intent identification

In natural language processing (NLP), as applied to chatbots, intents serve as a representation of the user’s specific intention, encompassing their desired outcome or the goals they aim to accomplish through their interaction. By recognizing these intents, NLP systems acquire a more nuanced and accurate analysis of the user’s interactions with the conversational agent [24] and consequently provide more accurate and contextually relevant responses which enhance the overall user experience [25–27].

Accordingly, we aimed to identify and categorize the various intents that could be derived from our dataset of userquestions. For example, when a user sought information regarding the symptoms associated with COVID-19, we categorized such a query as an “informational” intent, since it involves the acquisition of factual knowledge. Similarly, when a user inputs the simple phrase “Hello”, that was appropriately classified as”greeting” *intent*, denoting the user’s expression of salutations or opening remarks.

The classification of intents served to categorize the diverse range of user requests encountered in our questions dataset. This intent identification expanded the conversational capabilities of the chatbot, allowing it to cater to a wider array of user needs. To train the chatbot to understand user intents, the questions and answer pairs were each analyzed to derive the intent behind it. That generated approximately 535 distinct intent concepts (expanded from an initial 505), some implemented in both English and Luganda, which were used to train the language model to recognize similar themes and patterns and respond accordingly.

### Entity identification

We further strengthened our chatbot with the use of entities. In NLP, “entities” refer to the specific pieces of information (subjects and objects) that can be extracted from a text to provide context or detail. These are often nouns or noun phrases that hold particular significance within a sentence or larger text corpus. Entities can include a wide range of items such as names of people, organizations, locations, expressions of time, quantities, monetary values, percentages, and more. For example, if a user asks, “What are the symptoms of malaria?”, the chatbot needs to recognize “malaria” as an entity representing a disease, and then provide information related to the symptoms of malaria. In our case, we manually identified and collected entities related to the health domain such as diseases, locations, symptoms, and others to ensure that the chatbot could recognize and use those entities while forming responses. Entities were derived from multiple sources: FAQs provided health-related terms such as symptoms and conditions, while supplementary materials like Wikipedia contributed contextual entities including personal names, districts, and locations. This generated a total of 1,290 entities (Table 2). These manually curated entity lists were then used to train the automatic entity extraction components described below (Table 4).

**Table 4:** Initial Entities: The table above displays the types of entities in the dataset; entities are parts of speech that represent subjects like locations and names in the data.

No	Entity type	Examples	Count
1	Location entities	Kampala, Jinja, Mulago	119
2	Name entities	John, Eric, Atwine	1,122
3	Symptom entities	Headache, flue, fever	28
4	Diseases entities	COVID, HIV, Influenza	21

**Fig 2:**
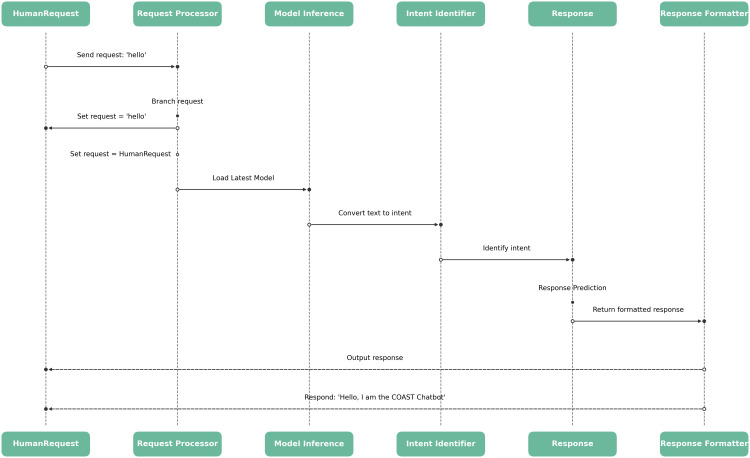
The conversation logic flow showing what happens from intent detection to text response in any conversation.

These were achieved by using space-based tokenizers, since both English and Luganda tokens are typically written with spaces in between them. This approach easily identifies and separates the different tokens within a request. For example the statement “What is covid?” has spaces between the three tokens that express the intent. The same is applicable in Luganda; “*Covid kye ki*?” is space delimited [28]. To accurately extract entities from user messages, we deployed a featurizer that is capable of creating lexical and syntactical features. This helped to effectively identify and categorize different entities within the input text [29]. That entity extraction was further enhanced using a built-in entity extractor in the DIET intent classifier to improve the precision. We also experimented with other entity extraction options to customize our pipelines for specific use cases. As an optional addition, we also made use of a pre-trained language model (Spacy) to estimate its effectiveness in our use case [18]. We reviewed the results of this trial and found that it provided useful insights into how our system could be further improved for example, we were able to determine how the size of the classifier was highly related to the accuracy of classification, i.e., the bigger the classifier (Spacy model) the better the accuracy but this came along with other deployment constraints. Ultimately, we chose to use the DIETClassifier (Dual Intent Entity Transformer) as our primary algorithm for natural language processing. This deep learning model combines intent and entity recognition in a single neural network, making it highly efficient and accurate in distinguishing user inputs and providing relevant responses. Its transformer-based architecture has been shown to outperform other traditional approaches such as rule-based systems and statistical models [30]. Furthermore, the DIET Classifier is capable of handling multiple intents and entities within a single sentence, making it particularly well-suited for complex dialogues that may arise in healthcare contexts. Finally, to model how these languages are used in real life, we used the built-in natural language understanding (NLU) component as developed by Rasa an open-source machine learning framework that enables AI assistants to interpret user messages by recognizing their intent and extracting key entities [31]. Through the implementation of stories, we modeled how the intents, entities, and other components of the conversation fit together to form a cohesive and effective dialogue. In this context, stories are example conversations that guide the NLU as to how different entities and intents should be sequenced within a conversation to form a coherent dialogue (Fig 2).

### Innovating for multilingual capability

To enable the chatbot’s bilingualism, we trained a classifier to effectively identify both English and Luganda intents and entities from within user messages. We employed an NLP approach based on the Rasa framework [30]. The Rasa framework, typically implemented in English, employs rule-based functionality to manage NLP tasks. The framework maps user intents to appropriate corresponding responses in a structured format which involves defining specific intents and their associated responses. Thus, once Rasa identifies an intent, it associates it with an appropriate response from the data mappings.

To extend that functionality to support interactions in Luganda, Rasa’s intent-response mapping was adapted and extended to incorporate Luganda language constructs. Specifically, a Luganda version of each English intent name was generated by appending ‘*_Luganda’* to that name, e.g., *greeting, greeting_Luganda, goodbye, goodbye_luganda.* Similarly, the corresponding values (potential responses) for each Luganda intent were mapped to it as the Rasa framework does for English intents. Furthermore, the Rasa framework was trained with thousands of English and Luganda sentences (queries) as examples and their corresponding derivative intents. That way, the model can predict the appropriate intent from English or Luganda user input queries from the Rasa mapping. That innovation enabled the chatbot’s bilingualism - depending on the language of the identified intent, the chatbot can seamlessly switch between English and Luganda.

### Pipeline development

The NLP framework that we constructed comprised a series of essential elements, meticulously chosen and refined to achieve precise identification and classification of intents and entities in both English and Luganda languages. In our initial approach, we employed the Whitespace Tokenizer which splits user messages into individual words based on inter-word spaces [32]. This step allowed us to break down the text into smaller units, facilitating further analysis and processing [33]. Following that tokenization process, we employed the RegexFeaturizer, which identifies patterns in these words (such as capitalization, punctuation, and word structure) to help the model recognize entities and understand sentence structure. This step significantly enhanced our understanding of the text by capturing important patterns and structures present in the message. By leveraging the power of regular expressions, we were able to extract relevant entities and gain deeper insights into the text. To augment that entity extraction capability, the CRFEntity Extractor was used [34]. This advanced technique identified and extracted specific entities from the text with greater accuracy. By employing the Conditional Random Fields (CRF) algorithm, we were able to train a model that could recognize and classify entities based on the context and surrounding words [35].

**Fig 3:**
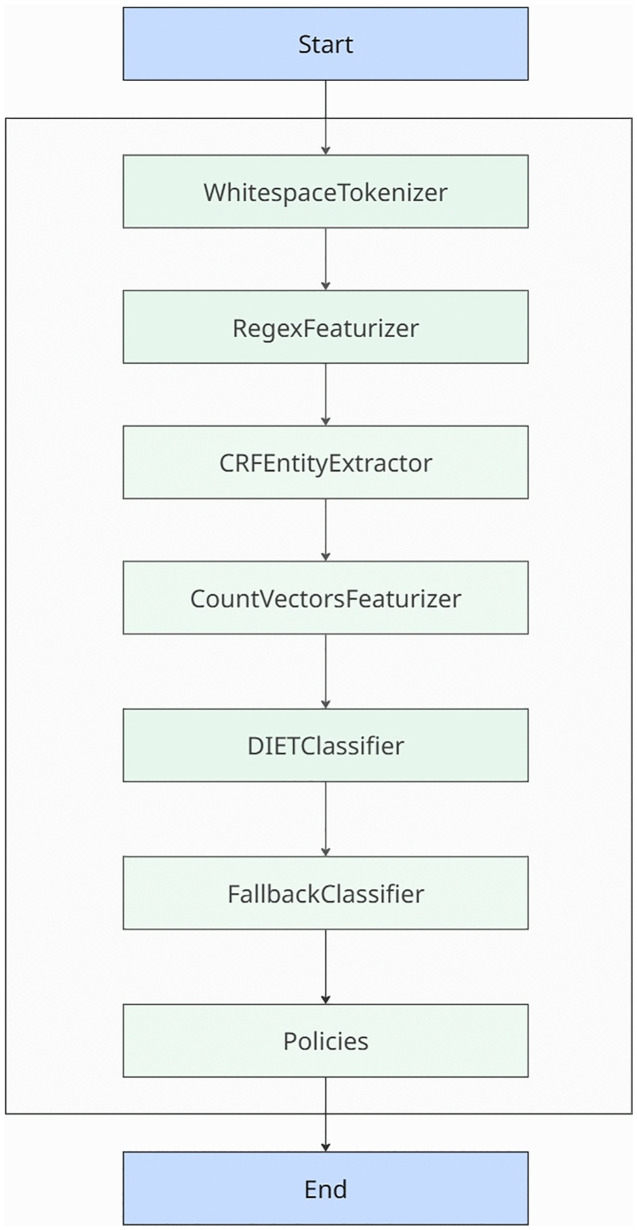
The technical flow and interconnectedness of the message processing components of the chatbot pipeline. User messages are tokenized (split into words), then processed through multiple featurizers that extract different patterns: RegexFeaturizer identifies word structure patterns, CountVectorsFeaturizer captures word and character sequence patterns (helping recognize common phrases and spelling variations), and CRFEntityExtractor identifies entities like locations and disease names. The DIETClassifier uses all these features to predict user intent and generate appropriate responses.

Overall, the approach involved a series of steps aimed at effectively processing and analyzing the given text. By utilizing the WhitespaceTokenizer, RegexFeaturizer, and CRFEntityExtractor, the pipeline achieved a comprehensive capacity to understand and extract meaningful information from input text (Fig 3).

Our experimentation, employed two instances of the CountVectorsFeaturizer. The first instance incorporated a minimum n-gram value of 3 and a maximum n-gram value of 6, allowing us to capture a wider range of linguistic patterns. An n-gram is a contiguous sequence of *n* items from a given sample of text or speech. An n-gram of size 1. For example, in the phrase “natural language processing,” the unigrams are “natural,” “language,” and “processing.” Concurrently, the second instance utilized a character analyzer with a minimum n-gram value of 2 and a maximum n-gram value of 4, enabling us to delve deeper into the intricacies of textual composition. These selected featurizers played a pivotal role in capturing supplementary attributes, thereby augmenting the overall precision and efficacy of our model.

To train our model, we used iterative experimentation resulting in the training of the DIETClassifier across 500 epochs, utilizing a learning rate of 0.0001 and an attention drop rate of 0.1. The training data was split using Rasa’s stratified random sampling at the example level (80% train, 20% test, random_seed = 42). This approach does not explicitly prevent paraphrases from appearing in both sets, which may moderately inflate performance estimates. Additionally, we incorporated four instances of the ResponseSelector, each trained over 450 epochs, with distinct retrieval intents. To ascertain the robustness of our model and its capability to process diverse input messages, we incorporated a FallbackClassifier into the system. The FallbackClassifier was configured with a threshold value of 0.2, ensuring that it would activate when the model’s confidence level fell below that threshold. The ambiguity threshold was set at 0.3, which determined the extent of uncertainty in the model’s predictions. We compared this DIET+CRF pipeline to the CRF-only pipeline and found that this DIET+CRF configuration shows statistically significant improvements over CRF-only (p < 0.001, Cohen’s d = 9.29), excellent generalization (train-test gaps < 1.1%), and high stability across cross-validation folds (CV < 0.03%). The Policies component handles dialogue management, determining the appropriate next action based on conversation context and history. Our implementation employed two complementary policies: the RulePolicy and the TEDPolicy (Transformer Embedding Dialogue Policy). The RulePolicy enforces deterministic conversation paths for structured interactions, such as greeting sequences and FAQ responses, ensuring consistent behavior for predictable user flows. The TEDPolicy, trained over 100 epochs with a maximum history of 8 dialogue turns, handles more dynamic conversations by learning patterns from training stories and generalizing to novel dialogue states. When both policies predict an action, Rasa’s policy priority mechanism resolves conflicts, with RulePolicy given higher priority (priority = 3) for rule-defined paths. This dual-policy approach balances the reliability of rule-based responses for common scenarios with the flexibility of machine learning for handling varied conversational patterns.

### Model deployment and serving

The chatbot we developed offers text interactions through a chatbot interface. This system’s architecture consists of two primary components: a user-facing frontend and a data processing backend. The front end, built using the React JavaScript library, stands as the main point of user interaction. It is structured with modular components like the chat window and message input area. The front end communicates with the backend using secure encryption protocols, enabling the secure exchange of user inputs and chatbot responses.

Our choice of React was driven by its component-based architecture, promoting modularity and efficient state management. This structure is ideal for a chatbot interface, allowing elements like messages and buttons to be individual components. React’s use of a virtual Document Object Model (DOM) ensures rapid and smooth updates, vital for a dynamic chat interface. Additionally, React’s flexibility enables integration with various backends, and its scalable component structure supports future expansion. The rich ecosystem of React, including its libraries, tools, and community support, further enhances the chatbot’s functionality.

On the other hand, the chatbot’s backend, built on the Flask Python framework, functions as the chatbot’s brain. It features diverse endpoints, such as the Chat Logic Processor for handling user inputs and generating responses. The system design also features secure and user-friendly authentication. We integrated Google’s OAuth 2.0 authentication, enhancing security and offering a familiar login interface to users. This not only bolsters security but also fosters user trust in the system.

### Beta testing

Since we wanted to create a chatbot that could effectively engage with users, we performed beta testing in two phases. The first phase was for preliminary user testing to ascertain the performance of all intended chatbot functions such as the NLU and NLP components as well as entity recognition and classification. For example an input with “John” should be classified as an entity “name”. On the other hand, the second phase sought to acquire insights into user experience.

### Phase one

Given the importance of capturing realistic conversation data, we employed Conversation Driven Development (CDD). This methodology emphasizes real-world interactions to refine and enhance chatbot conversational capabilities. For this phase, the chatbot was hosted on our on-premise nodes and testing was conducted in English owing to our design having it as the base language due to its more readily available resources. The prototype was made accessible to a diverse group of individuals - 41 males and 21 females including IDI health practitioners and trainers, software developers, medical interns as well as the general public for two weeks.

### Phase two

Having integrated changes arising out of insights collected from the first testing phase, we embarked on a second round of testing. It was largely focused on user experience - utility, user-friendliness, response speed and availability of the chatbot, rather than the nature, flow and themes/intents of questions and responses. Conducted in both English and Luganda, this phase included a broader linguistic scope (medical students, computer science students, IDI health and administrative workers) and an enhanced chatbot framework. Approximately 91 persons based on the unique conversation data recorded tested the chatbot for 14 days.

## Results

### Phase one results

Over this testing phase, there were 135 unique conversations with the chatbot. Each conversation represented an individual interaction with the prototype in which the taster asked the chatbot a series of questions of their choice. This extensive interaction provided insight into the common themes covered by the questions as well as the styles, nature and flow of questions. The conversations yielded 721 unique sentences from which we inferred the nuances, needs and expectations of user queries. To wit, approximately 30 new intents were identified and subsequently added to our intents database. These were additional unique user goals beyond the initial 505 intents (Table 5), which counted unique intents across both languages combined. This expansion significantly enhanced the chatbot’s ability to understand and respond to a broader range of user queries. Furthermore, some of the chatbot’s responses were perceived as lengthy, indicating a need to refine and condense responses into shorter answers for better legibility. Finally, there was a discernible difference between our initial assumptions of user queries and the actual questions posed during the trials. Key discrepancies included: (1) users asked more practical logistics questions than anticipated (e.g., “Where is the nearest testing center open on weekends?” rather than just “Where can I get tested?”), (2) significant interest in vaccine-specific concerns that we had underestimated (safety profiles, interactions with other medications, scheduling second doses), (3) more emotional/reassurance-seeking queries than our FAQ-focused approach had prepared for (e.g., “Am I going to die if I have COVID?”). These insights led to expanding both the intent coverage and the conversational flexibility of the chatbot. This highlighted the importance of continuous learning and adaptation based on real-world interactions (Table 5).

**Table 5:** Summarizes the outcomes of beta testing; the new information collected and the new data points it yielded.

Events	No of instances	Description
Full Conversations	135	The number of unique conversations with the chatbot
Natural Language Understanding (NLU) Data	721	New data points extracted from the conversations and intents
New Intents	30	New intents from which we curated from new data.

Following the feedback, insights and lessons learned from this initial testing, appropriate improvements were implemented on the prototype. These included revising the content of the answers to cover the additional themes/intents derived from the first phase, moderating/reducing the length of responses, integrating a more realistic dataset to capture the actual use cases of the target audience and implementing the Luganda component of the tool to extend the tool’s linguistic reach to our target audience.

### Phase two results

From this qualitative testing, we derived insights not just about the chatbot’s effectiveness, but also about user behavior and preferences. For example, it was clear that the dual-language capability catered to a wider demographic and ensured that language was not a barrier to information access. Sixty-four participants chose to interact in English, while 27 opted for Luganda, highlighting the importance of multilingual support. Overall, the second phase revealed the prototype as having higher utility owing to its bi-lingual capability, improved response times due to optimization of response text length, improved intent prediction and higher uptake as demonstrated by a higher number of testers.

### Intent identification, response accuracy and Bi-lingual capacity

**Fig 4:**
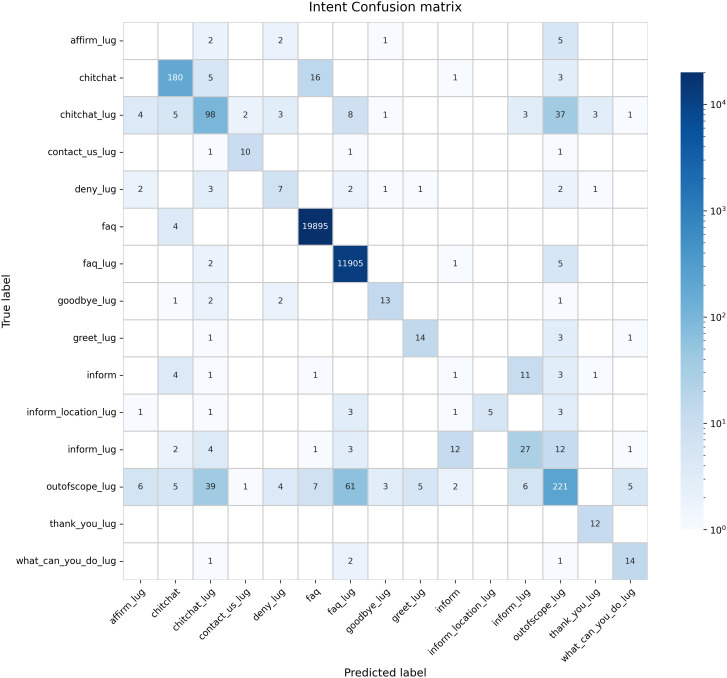
Intent classification confusion matrix showing how well the correct intent label was predicted for each query. The numbers on the chart are prediction values indicating the number of times a predicted label matched with the true label (on the middle diagonal) and the false label (if outside the middle diagonal).

Recognizing and abstracting user intents from queries is a critical element in ensuring the validity and usefulness of chatbot responses. Accordingly, the relevance of responses was measured by an assessment of the accuracy of intent prediction that the prototype was able to achieve. Using rigorous training with common questions and answers, the chatbot NLP algorithm acquired the ability to identify and differentiate various intents from within queries posed to it (Fig 4). Such intents included greetings, informational inquiries, disease descriptions, and conversation-ending. To validate this we performed a full cross-validation, whose results show low variance (std < 0.001), and the substantial weighted-macro F1 gap (98.87% vs. 62.01%). The chatbot accurately identified user intentions and generated appropriate responses. Out of a total of 13222 queries, the prototype predicted the intents in 12795 queries correctly and only 427 intents wrongly (Table 6). This represents an overall intent classification accuracy of 96.77%. We sought to check if the accuracy of these intent predictions is statistically significant using the Binomial test. The accuracy was statistically significant in English and in Luganda (Table 7) The prototype can therefore effectively and accurately respond to diverse types of inquiries initiated by users in either language. Nonetheless, we performed analysis to determine relative error rates for the major intents in either language (Table 6) which reveals the Luganda intents as more brittle despite remaining significantly accurate. Due to the inherent imbalance observed in the data with FAQs being far more, we checked the impact of the imbalance, using the Macro metrics test which gives equal importance to every intent, regardless of frequency. It revealed that indeed the rare events are predicted with less accuracy (F1 0.645).

**Table 6:** Intent classification performance metrics showing: (A) precision, indicating the relative number of correct vs incorrect intent predictions, (B) recall, showing the proportion of intents called accurately, (C) F1 score representing the harmonic mean of precision and recall, and (D) the count of each intent in the test data.

Intent	Language	Samples	Errors	Error_Rate_%	F1_Score
chitchat	English	205	25	12.19	0.88
chitchat	Luganda	165	67	40.61	0.60
faq	English	19,899	4	0.02	0.99
faq	Luganda	11,913	8	0.06	0.99
inform	English	22	21	95.45	0.05
inform	Luganda	62	35	56.45	0.49

**Table 7:** Performance evaluation of intent classification showing recall, F1-score, 95% confidence intervals, and statistical significance for each intent category. Sample sizes (n) vary by intent, with FAQ intents showing the highest sample sizes and performance. Intents suffixed with “_lug” indicate Luganda language utterances.

Intent	Language	n	Recall	F1	95% CI	p-value
faq	English	19,899	0.99	0.99	[0.99, 0.99]	<1e-300
faq_lug	Luganda	11,913	0.99	0.99	[0.99, 0.99]	<1e-300
chitchat	English	205	0.87	0.89	[0.83, 0.91]	3.2e-181
outofscope_lug	Luganda	365	0.61	0.67	[0.55, 0.65]	1.2e-158
chitchat_lug	Luganda	165	0.59	0.60	[0.52, 0.67]	9.0e-71
what_can_you_do_lug	Luganda	18	0.78	0.70	[0.55, 0.91]	8.1e-14
greet_lug	Luganda	19	0.74	0.72	[0.51, 0.88]	2.9e-13
goodbye_lug	Luganda	19	0.68	0.68	[0.46, 0.85]	9.5e-12
contact_us_lug	Luganda	13	0.77	0.77	[0.49, 0.92]	4.1e-10
thank_you_lug	Luganda	12	1.00	0.83	[0.76, 1.00]	7.7e-15
inform_lug	Luganda	62	0.44	0.49	[0.32, 0.56]	4.8e-16
inform_location_lug	Luganda	14	0.36	0.53	[0.16, 0.61]	0.0016
deny_lug	Luganda	19	0.37	0.38	[0.19, 0.58]	1.4e-04
inform	English	22	0.05	0.05	[0.01, 0.22]	0.78
affirm_lug	Luganda	10	0.00	0.00	[0.00, 0.28]	1.00

### Entity identification

The chatbot was designed to extract entities related to the health domain, such as diseases, locations, drugs, time and persons. We evaluated the entity extraction performance of the chatbot by testing it with queries containing specific entities. The chatbot accurately identified and utilized the extracted entities to provide relevant and tailored responses (Table 8).

**Table 8:** Entity extraction performance metrics showing the F1 scores for different entity types (locations, names, symptoms, diseases) in both English and Luganda. Higher F1 scores indicate better performance in accurately identifying and extracting specific entities from user queries.

Language	Entity	Precision	Recall	F1	Support
English	disease	0.97	0.98	0.99	19,679
English	symptom	1.00	1.00	1.00	56
English	name	0.56	0.35	0.43	40
English	location	0.88	1.00	0.93	14
Luganda	Obulwadde (disease)	0.97	0.99	0.98	10,624
Luganda	Erinya (name)	0.44	0.24	0.31	33
Luganda	Omulimu (profession)	1.00	0.09	0.16	22

### Key themes

**Fig 5:**
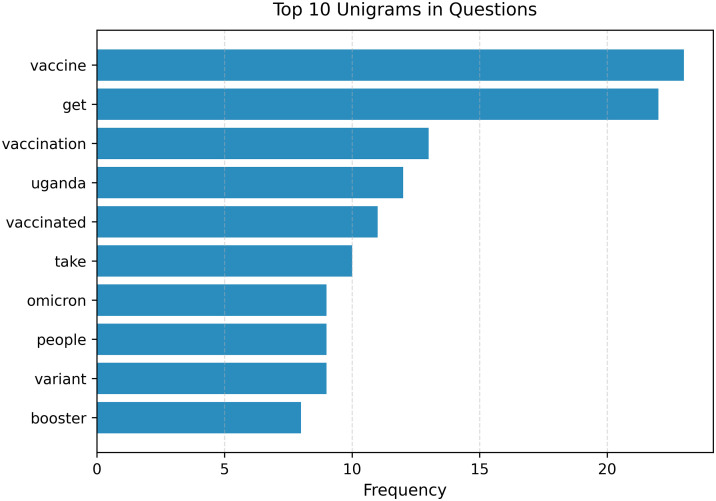
The figure shows a bar graph showing the most dominant English conversation themes from the beta testing.

Several themes emerged from the conversations (Fig 5), with the top themes captured by users’ concerns and information queries being about: vaccination, COVID variants, treatments, and symptoms, as well as a strong focus on vaccine efficacy, prevention strategies, and the Omicron variant.

### Chatbot prototype

**Fig 6:**
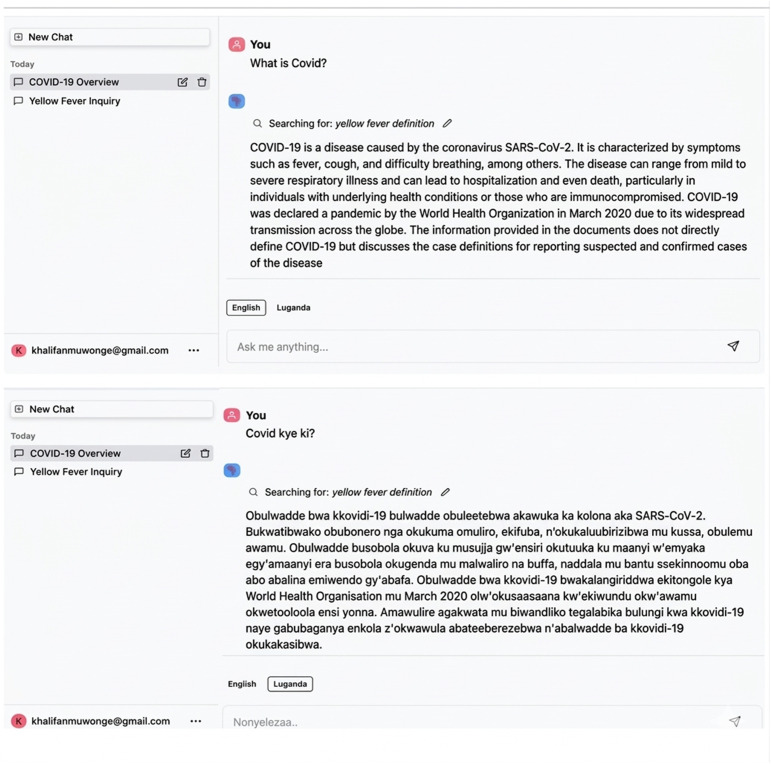
The chatbot frontend shows the simple look and feel of the chatbot user page; new chats can be started at the top left corner, and the main panel is where the chats happen through the input text box at the bottom in English or Luganda respectively.

The back-end components, which included the trained models, were integrated into the Rasa framework. That framework is itself built on a micro services architecture which coordinates the cross-talk between the databases, the models and the user interactions on the front-end. Those aggregated components were then deployed onto a Kubernetes-based platform to allow real-time scaling as the volume of usage fluctuates. This entire setup constituted the functional prototype (Fig 6).

**Impact** This innovative approach not only leveraged Rasa’s rule-based NLP processing framework, but it also extended its functionality to meet the unique linguistic requirements of our use case. Consequently, the chatbot; 1) Recognizes and processes user inputs in Luganda by mapping Luganda intents to corresponding responses and 2) Responds appropriately in either English or Luganda, depending on the user’s input language. That enables seamless switching between languages as and when a user so desires.

This approach demonstrates how existing technological frameworks can be innovatively utilized to address specific linguistic NLP needs, thereby enhancing the reach of such technologies.

## Discussion

In this work we sought to bring affordable language technology to bear in a low-resource setting, in the context of pandemic management, which was particularly susceptible to misinformation. Chatbots have the potential to mitigate misinformation in pandemic settings. This work constitutes the development of a chatbot model using natural language processing techniques. We employed the DIET classifier, a deep learning model based on transformers, which has been shown to outperform traditional approaches [30]. The DIET classifier combines intent and entity recognition in a single neural network, making it highly efficient and accurate in understanding user inputs and providing relevant responses. This model serves as a robust foundation for building chatbots that can handle complex dialogues and deliver accurate information.

Such chatbots retain special importance even in light of large language models (LLMs) that have become increasingly mainstream in the years since this work started in 2020. Although less conversational in comparison to LLM-driven platforms, they are less susceptible to hallucination (the generation of plausible-sounding but factually incorrect or unsupported information) since their responses are drawn from only the curated data used to build them. They also remain less expensive to build as they can be trained with little or no GPU time. This fact is of important consequence since GPUs or GPU time for model-training is expensive to purchase whether in the cloud or on-premises and can be a real limitation in low-resource settings.

Yet another major contribution of this work is the leveraging of transferable logic techniques to enable the identification of Luganda intents using pre-defined mappings of English logic functionalities. That innovation is what enabled the bi-lingual capabilities of the prototype at an affordable cost and with relative ease. This contribution promotes language inclusivity by making language technology accessible to low-resourced languages that are widely spoken in specific regions, like Luganda in Uganda. While improving information access through enabling Luganda, this chatbot remains subject to the gap between the written word and spoken language. The absence of Text-to-speech (TTS) and Speech-to-text (STT) capabilities from its tool kit leaves room for improvement towards a more interactive and accessible user experience. That constitutes future work which, although challenging given the bilingual nature of the chatbot, will be potentially impactful for the same reason.

Regarding the imbalance in our dataset - FAQ intents constitute a disproportionately higher number of queries and thus introduce a measure of inaccuracy in the predictive model. However, frequency alone did not determine classification accuracy. The model achieved near-perfect performance on infrequent intents such as “affirm” (1.00 precision and recall with minimal training examples) and “what_can_you_do” (0.91 F1-score), while “inform_location” reached 0.76 with comparable data availability. These variations suggest that intent separability in the feature space played a more critical role than training set size. Conversely, some frequent intents like “goodbye” (0.56 F1-score) exhibited confusion with semantically related intents such as “thank_you” and “greet,” indicating that lexical overlap between conversational closings posed classification challenges regardless of training volume. This pattern underscores the importance of semantic distinctiveness in intent classification, where well-defined intent boundaries enable accurate recognition even with limited examples, while ambiguous boundaries can challenge the model despite abundant training data.

The chatbot has scalable and long-term deployment potential due to its modular and open-source architecture. Rasa’s components—such as NLU (Natural Language Understanding), Core (dialogue management), and custom action servers—can be containerized using tools like Docker and orchestrated with Kubernetes for load balancing and horizontal scaling. This makes it feasible to serve a growing number of users across both languages without sacrificing performance. Additionally, Rasa supports on-premise and cloud deployments, allowing adaptation of infrastructure configurations when need arises. With appropriate model retraining, and version control, the chatbot can evolve seamlessly as new intents, entities, and language variations emerge, ensuring sustainable long-term performance. Rasa’s self-hosted nature provides control over data handling. Sensitive user inputs remain within the infrastructure, minimizing exposure to third-party systems and thereby facilitating security and privacy. Finally, this being a multi-lingual chatbot, entity disambiguation is important and was achieved to statistically significant levels that achieved accurate, secure, and contextually aware responses in both languages as seen in (Table 7).

Taken together, this work, at least in part, illustrates how, with appropriate innovation, low-resourced languages can be supported by more affordable technologies that leverage pre-existing resources of highly resourced languages. It also supports the notion that despite the recent maturation of LLMs, there remains a niche that can be filled by a hybrid of Machine Learning and rule-based natural language technology.

### Outputs bilingual, prototype, data collection & protocol, 24/7 availability, language technology

By using this data, other researchers and developers can train and evaluate their chatbot models, fostering further advancements in the field. This dataset is domain-specific in building bots that are related to pandemics.

### Code availability

The underlying code for this study is available in this GitHub repository: https://github.com/aceuganda/idi_faq_mvp
